# Molecular detection of tick-borne pathogens in captive wild felids, Zimbabwe

**DOI:** 10.1186/s13071-014-0514-6

**Published:** 2014-11-18

**Authors:** Patrick Kelly, Lisa Marabini, Keith Dutlow, Jilei Zhang, Amanda Loftis, Chengming Wang

**Affiliations:** Ross University School of Veterinary Medicine, Basseterre, St. Kitts and Nevis; AWARE Trust, Harare, Zimbabwe; Jiangsu Co-innovation Center for Prevention and Control of Important Animal Infectious Diseases and Zoonoses, Yangzhou University College of Animal Science and Technology, Yangzhou, Jiangsu 225009 China; Department of Microbiology & Immunology, Midwestern University, Glendale, AZ USA

**Keywords:** *Babesia*, *Ehrlichia*, *Anaplasma*, Wild felids, Zimbabwe

## Abstract

**Background:**

The populations of wild felids in Africa, of especially lions (*Panthera leo*) and cheetahs (*Acinonyx jubatus*), are declining and the species are classified as vulnerable to extinction by the International Union for Conservation of Nature. As infections with tick-borne pathogens (TBP) can become more of a problem in wild felids, there are relatively few studies on TBP in wild felids in Africa and on how these infections might influence population numbers.

**Methods:**

To gain further knowledge on TBP in captive wild felids in Southern Africa, we collected whole blood from captive lions, Southern African wildcats, cheetahs and servals in Zimbabwe for PCRs against the 18S rRNA gene of the piroplasmids (*Babesia, Theileria, Cytauxzoon*) and *Hepatozoon* spp., and the 16S rRNA gene of *Ehrlichia* and *Anaplasma* spp.

**Results:**

Overall, 78% of the lions (67/86) and all the Southern African wildcats (6/6), cheetahs (4/4) and servals (2/2) had evidence of infection with at least one organism. The organisms most commonly detected in the lions were *B. leo* (59%; 51/86), *B. vogeli* (12%; 10/86) and *H. felis* (11%; 9/86) while all the Southern African wildcats and servals were positive for *B. vogeli* and all the cheetahs were positive for *B. leo*. Mixed infections were found in 22% (15/67) of the PCR positive lions, most commonly *B. leo* and *H. felis* (27%; 4/15), and in 1 (50%) of the servals (*B. vogeli* and *A. phagocytophilum*). Two lions were infected with three TBP, mainly *B. leo, H. canis* and *T. parva*, and *B. leo*, *A. phagocytophilum* and *T. sinensis*. Mixed infections with *B. vogeli* and *A. phagocytophilum* were seen in a serval and a Southern African wildcat. Other TBP were detected at a low prevalence (≤2%) in lions, mainly *H. canis*, *T. sinensis, T. parva*, *C. manul, E. canis, and E. canis*-like and *B. odocoilei*-like organisms.

**Conclusions:**

Infections with tick-borne agents are common in captive wild felids in Zimbabwe.

## Background

The populations of wild felids in Africa, especially lions (*Panthera leo*) and cheetahs (*Acinonyx jubatus*), are declining and the species are classified as vulnerable to extinction by the International Union for Conservation of Nature (http://www.iucnredlist.org, accessed 21^st^ July 2014). Similarly, although figures are not available, the Southern African wildcat (*Felis silvestris cafra*) is thought to be endangered due to hybridization with domestic cats and contracting their infectious diseases [1]. Human expansion has resulted in loss of natural habitat for the endangered wild felids which are increasingly being incorporated into wildlife reserves or parks where they play an important role in ecotourism, a major source of revenue for many African countries. In these smaller territories, ectoparasites can become more of a problem and this may increase the possibility of infections with tick-borne agents [2].

There are relatively few studies on TBP in wild felids in Africa and thus little information on how these infections might influence population numbers. The earlier studies in which parasites were identified microscopically [3,4] are of limited value, as many TBP are difficult to detect in blood films and it is not generally possible to determine the species of pathogens by morphological criteria. More recent studies using molecular methods have provided greater details on the agents that infect lions and cheetahs but only five have been carried out to date describing the situation in 436 lions and 142 cheetah in seven countries: 81 lions from South Africa and Swaziland and 137 cheetah from South Africa and Namibia [5], 21 lions in Botswana [6], 301 lions in Tanzania [7], 9 lions and 5 cheetah in Kenya [8] and 24 lions in Zambia [9]. To add to this current knowledge, we studied TBP in lions, Southern African wildcats, cheetahs and serval (*Leptailurus serval*) from Zimbabwe and describe our findings below.

## Methods

### Whole blood samples

Whole blood samples (n = 98) were collected into EDTA from apparently healthy captive lions in the towns of Gweru (n = 67) and Masvingo (n = 6) and Dollar Block farm (n = 8). They were also collected from captive Southern African wildcats (n = 6), cheetahs (n = 4) and servals (n = 2) in the city of Harare. Five further lions were sampled in the town of Gweru, within days of being translocated from the town of Hwange (n = 3) and the village of Fig tree (n = 2) where they had been kept in captivity. All animals in the study were over 6 months of age and all had been in captivity for at least 6 months. They were apparently healthy and were sampled while being anesthetized for microchipping and routine health monitoring or procedures. The study was reviewed and approved by the Institutional Animal Care and Use Committee of the Ross University School of Veterinary Medicine (RUSVM), St Kitts.

### DNA extraction and quality control

After collection, blood samples were frozen in Zimbabwe at -20°C before being sent on ice to RUSVM where DNA was extracted from aliquots (200 μl) using the QIAamp DNA Blood Mini Kit (Qiagen, Valencia, CA, USA) according to the manufacturer’s instructions. The DNA was eluted in 200 μl elution buffer and shipped to Yangzhou University College of Veterinary Medicine of China at room temperature where it was frozen at -80°C until PCRs were performed.

### PCRs

The PCRs were performed on a Roche LightCycler 480-II platform. The HMBS gene was used as an endogenous control [10] and a conventional PCR was used to detect a 848-bp fragment of the 18S rRNA gene with previously described primers (BTF1 - forward: 5′-GGCTCATTACAACAGTTATAG-3′; BTR2 - reverse: 5′-GGACTACGACGGTATCTGATCG-3′) [11]. These primers are reported to detect most species of *Babesia* and *Theileria*. To ensure the primers detected the feline *Babesia* spp., we used them in the BLASTN program to search the sequence data available on GenBank.

A conventional PCR was also performed to detect a 478 base pair fragment of the 16S rRNA gene of *Ehrlichia* spp. and *Anaplasma* spp. using primers (ECC - forward: 5′-AGAACGAACGCTGGCGGCAAGCC-3′; ECB - reverse: 5′-CGTATTACCGCGGCTGCTGGCA-3′) described previously [12].

Positive and negative controls were as described in the relevant references. For all PCRs, positive products were verified by gel electrophoresis and nucleotide sequencing using forward and antisense primers (GenScript, Nanjing, China).

## Results

The HMBS-based FRET-PCR showed average HMBS copy numbers in the 97 samples to be 3.5 ± 1.2 × 10^6^ copies/ml whole blood (range 4.0 × 10^4^ to 2.5 × 10^7^), indicating the DNA in each sample was of sufficient quality for further PCR studies. The BLASTN program showed the BTF1 and BTR2 primers had 100% identity with the 18S rRNA gene of three genera (*Babesia* spp., *Theileria* spp., and *Hepatozoon* spp.) and only a single base pair mismatch in the BTF1 with *Cytauxzoon* spp. The primers recognized all the feline *Babesia* spp. with 100% identity, except for *B. felis* where there was a single mismatch in the BTF1.

Following generally accepted rules of taxonomy [13], organisms we identified with sequences that were at least 97% identical to a recognized TBP on GenBank were regarded to be that species while those with lesser identity were regarded to be species-like. Sequences with under 100% identity and at least 200 base pairs in length were submitted to GenBank and the following accession numbers were obtained: KJ598879 - 94% identity with *E. canis*, KJ598888 - 97.6% identity with *E. canis*, KJ598889 - 99.4% identity with *A. phagocytophilum*; KJ598890 - 99.5% identity with *A. phagocytophilum*; KJ598891 – 99.3% identity with *A. phagocytophilum*, KJ598880 - 97.5% identity with *B. vogeli*; KJ598881 - 94.8% identity with *B. odocoilei*; KJ598882 - 94.6% identity with *B. odocoilei*; KJ598886 - 99.3% identity with *H. felis*; KJ598887 - 99.2% identity with *H. felis*; KM211712 - 99.5% identity with *T. parva*.

Overall, very high percentages of lions (78%; 67/86), Southern African wildcats (100%; 6/6), cheetahs (100%; 4/4) and servals (100%; 2/2) had evidence of infection with at least one of the TBP (Table 1). Each of the nine recognized TBP we found, as well as *B. odocoilei*-like and *E. canis*-like organisms, were present in the lions while only *B. vogeli* and *A. phagocytophilum* were found in Southern African wildcats and servals, and only *B. leo* in cheetahs. Overall, the most common TBP we detected in the animals we studied was *B. leo* (56%; 55/98) with *B. vogeli* (18%; 18/98) being the next most prevalent. Infections with a single organism were seen in 78% (52/67) of the PCR positive lions, mainly *B. leo* (38), *B. vogeli* (9), *B. odocoilei*-like (1), *H. felis* (3), or *C. manul* (1). Mixed infections were seen in 22% of the PCR positive lions (15/67) [*B. leo* and *H. canis* (1); *B. leo* and *E. canis* (1); *B. leo* and *E. canis*-like (1); *B. leo* and *H. felis* (4); *B. leo* and *A. phagocytophilum* (3); *B. leo*, *H. canis* and *T. parva* (1); *B. leo*, *A. phagocytophilum* and *T. sinensis* (1); *B. leo*, *A. phagocytophilum* and *H. felis* (1); *B. vogeli* and *H. felis* (1); or *B. odocoilei*-like and *C. manul* (1)]. A mixed infection with *B. vogeli* and *A. phagocytophilum* was seen in one of the servals and one of the Southern African wildcats, but there were no mixed infections in the cheetahs (Table 1).

**Table 1:** **Survey sites, animals tested and results of PCR analyses for tick borne pathogens**

**Species**	**Area**	**N**	***Babesia leo***	***Babesia vogeli***	***Hepatozoon felis***	***Hepatozoon canis***	***Anaplasma phagocytophilum***	***Theileria sinensis***	***Theileria parva***	***Cytauxzoon manul***	***Ehrlichia canis***
Lions	Gweru*	67	64% (43)	12% (8)	11% (7)	3% (2)	8% (5)	2% (1)	2% (1)	2% (1)	
	Dollar Block	8	63% (5)								12% (1)
	Hwange	3	33% (1)	67% (2)			33% (1)				
	Fig Tree	2	50% (1)		100% (2)						
	Masvingo	6	17% (1)							17% (1)	
	All lions	86	59% (51)	12% (10)	11% (9)	2% (2)	7% (6)	1% (1)	1% (1)	2% (2)	1% (1)
Southern African wildcats	Harare	6		100% (6)			13% (1)				
Cheetahs	Harare	4	100% (4)								
Servals	Harare	2		100% (2)			50% (1)				
**Totals**		**98**	**56% (55)**	**18 % (18)**	**9% (9)**	**2% (2)**	**8% (8)**	**1% (1)**	**1% (1)**	**2% (2)**	**1% (1)**

*Also present were 2 lions infected with *B. odocoilei*-like organisms and 1 with an *E. canis*-like organism.

## Discussion

The wild felid species we studied in Zimbabwe were commonly infected with *Babesia* with 73% (63/86) of the lions and all the Southern African wildcats (6), cheetahs (4) and servals (2) being PCR positive. All animals were apparently healthy at the time of blood collection, indicating a high prevalence of chronic subclinical infections. The most common TBP we identified was *B. leo* which we identified in 59% of the lions and all the cheetahs (4) we studied. *Babesia leo* is a small *Babesia* spp. which was first described in 2001 [14] and has been reported to be very common in lions (28%) in Southern Africa [5] but far less common in Tanzania (<1%) [7]. It has only been previously reported at low prevalence (0-3%) in cheetahs in Southern Africa. Phylogenetic analysis shows it forms a group with *B. felis* which infects domestic cats, *B. microti* which infects rodents and humans, and *B. rodhaini* which infects rodents. The vector of *B. leo* is unknown, but infected lions have been found to be commonly infested with *Amblyomma hebraeum, Haemaphysalis leachi* and *Rhipicephalus simus* [14]. While we did not study the ectoparasites on the animals we tested, local experience is that captive lions are more commonly infested with ticks while cheetahs have light tick burdens but more severe flea infestations. Tick and flea control products used by owners of wild felids in Zimbabwe include Deltatick Pour On® (Chemplex, Harare, Zimbabwe), a topical 5% deltamethrin product for use in cattle, and Frontline Spray® (Merial, South Africa), a fipronil based product reported to control fleas and ticks on dogs. To the best of our knowledge these products have not been evaluated for efficacy or safety in wild felids.

Although around 1% of stray domestic cats in Thailand have been found to be infected with *B. vogeli* [15] ours is the first report of the organism in Southern African wildcats (100%; 6/6), lions (12%; 10/86) and servals (100%; 2/2). *Babesia vogeli* commonly infects dogs in tropical and subtropical areas with prevalences of 4-60% [16]. The organism is transmitted by *R. sanguineus* and infections are mostly asymptomatic with dogs becoming subclinical carriers [17]. *Rhipicephalus sanguineus* was not found on Southern African domestic or wild felids in a recent survey which was regarded as consistent with the near-strict host preference of the tick for domestic dogs [2]. Further studies are needed to determine the transmission mechanisms in wild felids.

We found no evidence of *B. felis* in the wild felids we studied which was also the case in a study of 2 lions and 5 cheetahs from Kenya [8]. Nevertheless, infections have been described in lions and cheetahs, both free-living and captive, from Southern Africa (4-28%) [5] as well as in lions from Zambia (number not given) [9] and Tanzania (number not given) [7]. One infected serval has also been reported [18]. A possible reason we failed to detect the organism is because the BTF1 primer we used in our PCR had a mismatch with *B. felis* which might have reduced the sensitivity of the reaction and required relatively high copy numbers of *B. felis* to give positive reactions. Also, our detection system using a standard PCR and sequencing is not sensitive for mixed infections which are reported to be common using methods which are more accurate in their detection [5]. Investigations with more specific *B. felis* primers are planned to resolve this issue.

We also failed to identify the other *Babesia* spp. that have been described in wild felids in Africa. In Tanzania and Kenya, lions have been found to be infected with an organism most similar to *B. gibsoni* [7] and *B. canis* (98% identity) [8], respectively, both of which have previously only been described in dogs. *Babesia lengau* is a recently described species that was found in a high percentage (29%) of cheetahs in South Africa [19]. A closely related species has been found in spotted hyenas (*Crocuta crocuta*) and a lion in Zambia [9]. The organism appears to result in asymptomatic infections in cheetahs, but in domestic cats [20] and sheep [21], *B. lengau* and a closely related organism, have been reported to cause cerebral and hemolytic babesiosis, and hemolytic disease, respectively.

Undescribed *Babesia* spp. have been reported to be common in African wild felids [5] and we found an *B. odocoilei*-like organism in two lions. This large *Babesia* has, to date, only been described in North America in a variety of wild ruminants [22]. Recently seropositive rabbits have been reported [23] and the amplicons of PCRs for *Babesia* on two Florida panthers (*Puma concolor coryi*) had similar sequences [24]. Also, *Ixodes ovatus* from dogs in Japan have been found to contain *B. odocoilei*-like organisms (97.7% identical) [25]. It is of note that a large *Babesia* has been described in a domestic cat from Zimbabwe [26] and further studies are indicated to more clearly describe these novel *Babesia* spp.

Although *Cytauxzoon* spp. have been described by molecular studies in wild felids from US [27], Spain [28] and Brazil [29], ours is the first report of the organism in wild felids in Africa. Sequencing of the organism showed it was most similar to *C. manul* (99% identity) which is found in Pallas cats (*Otocolobus manul*) in Mongolia [30]. While a cytauxzoonosis-like disease has been described in a domestic cat in Zimbabwe [31], experimental infection studies using *C. manul* in domestic cats resulted in no clinical signs although there was a low parasitemia [32].

Our finding of *Hepatozoon* spp. in both lions and cheetahs was not unexpected as they have been seen frequently in blood smears from African wild felids [3,4]. There is little data, however, on their molecular biology with organisms being identified by molecular means in 38% of Zambian lions [9], an unspecified number in lions from Tanzania [7], and 55% of Asiatic lions [33]. The species identified in the Asian and Tanzanian lions was *H. felis* while *H. canis-*like organism were detected in the Zambian lions. There is considerable diversity in *Hepatozoon* spp. in wild canids in the US [34] and further studies are indicated to determine if this is also the case in African wild felids. Although generally regarded as being non-pathogenic in wild felids, there is a report of *Hepatozoon* infection perhaps contributing to the death of spotted hyenas (*Crocuta crocuta*) in Tanzania. The organism involved had a 18S RNA gene sequence identical to that of a *Hepatozoon* sp. described previously in domestic cats in Spain which is distinct from *H. canis* [35].

Although *Theileria-*like parasites have been seen in blood films of cheetahs from Tanzania [4] and South Africa [36] there has been only a single report of the molecular identification of organisms in African wild felids. With reverse line blots, *Theileria* spp. were found in cheetahs and a lion from Kenya with sequencing indicating the parasites were closely related to those infecting sheep and giraffes [8]. We had positive PCR reactions for two lions with sequencing showing the organisms were most similar to *T. parva*, the agent of Corridor disease and East Coast Fever in domestic ruminants, and *T. sinensis* that infects cattle and yaks in China [37]. Neither of these species has been described in felids and further genomic studies are required to more precisely characterize the organisms and determine their pathogenicity.

Six of the lions, one of the servals, and one of the Southern African wildcats we studied were infected with an organism most similar to *A. phagocytophilum*, a tick-borne rickettsia infecting a wide range of species including domestic and wild ruminants, horses, dogs, humans and domestic cats. It has been found in wild felids outside of Africa, including up to 10% of lions in Italy and California [38,39], but none of 21 free ranging lions in Botswana were seropositive [6]. Although widely distributed in the Northern Hemisphere where it’s *Ixodes* spp. vectors are common, in Sub-Saharan Africa *A. phagocytophilum* has only been described in three sheep in Senegal [40] and a closely related organism was reported in a dog in South Africa [41]. *A. phagocytophilum* can cause severe disease in animals and people and in domestic cats has been reported to cause fever, lethargy and inappetence with non-specific signs including tachypnea, muscle and joint pain, neurological signs and lymphadenomegaly that respond to tetracycline therapy [42-46].

*Ehrlichia canis* is the rickettsial agent of canine monocytic ehrlichiosis which is transmitted by the brown dog tick, *Rhipicephalus sanguineus.* Infected dogs are found very commonly around the world but infections in domestic cats are only reported infrequently. In many cases the cats are asymptomatic but signs including intermittent fever, weight loss, vomiting and diarrhea have been reported, as have thrombocytopenia and anemia [47]. Small numbers of wild felids infected with *E. canis* have been reported from Brazil [48,49] and Japan [50] and, although a serosurvey in Botswana [6] and a PCR survey in Zambia [9] were negative, there is evidence of *E. canis* infections in domestic cats in Southern Africa [51]. Our finding of two lions infected with *E. canis* or *E. canis-*like organisms is consistent with the available data showing infections are uncommon in felids generally. This may be related to the fact that the vector of *E. canis*, the brown dog tick *R. sanguineus*, very seldom feeds on species other than the domestic dog [2].

## Conclusions

In summary, our study has added to the knowledge of the tick-borne infections of wild felids in Southern Africa. Although further molecular studies are needed to more precisely determine the range of species involved, the available evidence is that infections with tick-borne agents are common, perhaps more particularly in captive animals where ticks can become more prevalent. Although most of the infected wild felids we tested appeared healthy and most likely had chronic infections, studies are needed to determine if there are signs associated with acute infections and if the tick-borne organisms can potentiate other conditions. Studies are also needed on optimal ways of controlling ticks in captive wild felids to control infections.
